# Laminin 332-functionalized coating to regulate the behavior of keratinocytes and gingival mesenchymal stem cells to enhance implant soft tissue sealing

**DOI:** 10.1093/rb/rbac054

**Published:** 2022-08-02

**Authors:** Lipeng Liu, Jing Wang, Ying Li, Bing Liu, Wei Zhang, Weikang An, Qing Wang, Boya Xu, Lingzhou Zhao, Chufan Ma

**Affiliations:** State Key Laboratory of Military Stomatology & National Clinical Research Center for Oral Diseases & Shaanxi Key Laboratory of Stomatology, Department of Prosthodontics, School of Stomatology, The Fourth Military Medical University, Xi'an, Shaanxi 710032, China; State Key Laboratory of Military Stomatology & National Clinical Research Center for Oral Diseases & Shaanxi Key Laboratory of Stomatology, Department of Prosthodontics, School of Stomatology, The Fourth Military Medical University, Xi'an, Shaanxi 710032, China; Department of Stomatology, Air Force Medical Center, The Fourth Military Medical University, Beijing 100142, China; Department of Stomatology, Air Force Medical Center, The Fourth Military Medical University, Beijing 100142, China; State Key Laboratory of Military Stomatology & National Clinical Research Center for Oral Diseases & Shaanxi Key Laboratory of Stomatology, Department of Prosthodontics, School of Stomatology, The Fourth Military Medical University, Xi'an, Shaanxi 710032, China; State Key Laboratory of Military Stomatology & National Clinical Research Center for Oral Diseases & Shaanxi Key Laboratory of Stomatology, Department of Prosthodontics, School of Stomatology, The Fourth Military Medical University, Xi'an, Shaanxi 710032, China; State Key Laboratory of Military Stomatology & National Clinical Research Center for Oral Diseases & Shaanxi Key Laboratory of Stomatology, Department of Prosthodontics, School of Stomatology, The Fourth Military Medical University, Xi'an, Shaanxi 710032, China; State Key Laboratory of Military Stomatology & National Clinical Research Center for Oral Diseases & Shaanxi Key Laboratory of Stomatology, Department of Prosthodontics, School of Stomatology, The Fourth Military Medical University, Xi'an, Shaanxi 710032, China; Department of Stomatology, Air Force Medical Center, The Fourth Military Medical University, Beijing 100142, China; State Key Laboratory of Military Stomatology & National Clinical Research Center for Oral Diseases & Shaanxi Key Laboratory of Stomatology, Department of Prosthodontics, School of Stomatology, The Fourth Military Medical University, Xi'an, Shaanxi 710032, China; Department of Stomatology, Air Force Medical Center, The Fourth Military Medical University, Beijing 100142, China

**Keywords:** PDLLA, laminin 332, keratinocytes, gingival mesenchymal stem cells, soft tissue sealing

## Abstract

Peri-implant epithelial sealing is the first line of defense against external pathogens or stimuli; hence, an essential process to prevent peri-implantitis. Laminin 332 (LN332) is the main component of the internal basal lamina and participates in peri-implant epithelial sealing by forming hemidesmosomes (HDs) with integrin α6β4. In this work, poly (D, L-lactide) (PDLLA)-LN332 composite coating was successfully constructed by a method similar to layer-by-layer assembly, displaying staged LN332 release for as long as 28 days. The PDLLA-LN332 composite coating can activate the intracellular PI3K-Akt pathway via binding to cellular integrin α6β4, which can promote adhesion, migration and proliferation of HaCaT cells and further enhance the expression of keratinocyte HD-related molecules, including integrin α6β4, LN332 and plectin. Furthermore, the PDLLA-LN332 composite coating can promote the adhesion, spreading and proliferation of gingival mesenchymal stem cells and accelerate their epithelial differentiation. Therefore, the PDLLA-LN332 composite coating can enhance implant soft tissue sealing, warranting further *in vivo* study.

## Graphical Abstract

**Figure:**
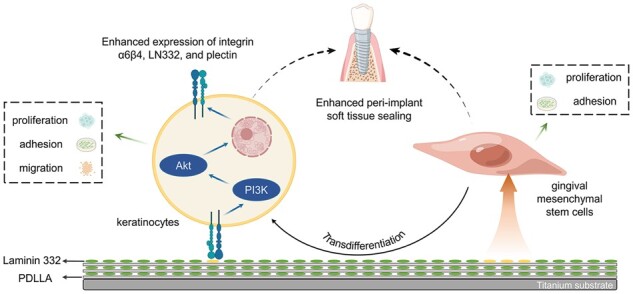

## Introduction

Titanium (Ti) and Ti alloys have been the material of choice for oral implants during the last few decades, owing to their good mechanical properties, corrosion resistance and biocompatibility [1]. However, implant failures still occur, possibly due to bacterial infection and weak soft tissue integration [2]. There is scarce research focusing on improving the soft tissue sealing compared to the abundant research on improving the osteogenic and antibacterial properties of Ti implants.

The soft tissue sealing consists of epithelial and connective tissue around natural teeth, and the epithelial sealing is considered to be the first line of defense against bacterial invasion [3]. The junctional epithelium (JE) connects to the enamel via hemidesmosomes (HDs) and basement membrane (BM)-like extracellular matrix (ECM) called the internal basal lamina (IBL) [2, 4]. HDs are multiprotein complexes composed of laminin 332 (LN332), integrin α6β4 and plectin. The same structures have been reported to exist between the peri-implant epithelium (PIE) and the implant surface [5]. LN332 is deposited in the wound bed by keratinocytes following implant placement, which mediates keratinocytes adhesion and migration [6, 7]. Furthermore, HDs and IBL predominantly reside at the apical portion of the interface between the implant and PIE, which may be associated with insufficient LN332 secretion and insufficient integrin α6β4 expression [8].

Some scholars have applied LN332 to the surface of Ti or zirconia to improve the implant–soft tissue interaction and have found that LN332 improved the proliferation and adhesion of keratinocytes and promoted HDs formation [9–12]. Following implant implantation, activated coagulation leads to the activation of the fibrinolytic system, resulting in LN332 degradation by plasmin [13]; thus, an enhanced loading amount and a controlled release of LN332 are required during implant–soft tissue wound healing for a stable and long-term function. Covalent binding may not be a good solution since a conformation change occurs during LN332 covalent binding to the substrate, which affects its function [11]. Poly(D, L-lactide) (PDLLA) is broadly applied in drug delivery systems and implant coatings due to its good biocompatibility and biodegradation properties and low ability to cause immune and allergic reactions [14]. Layer-by-layer assembly (LBL) is a pervasive method for surface coatings construction, requiring a polyelectrolyte initiator layer on the substrate and two macromolecular polymers with opposite charges [15]. PDLLA has been found to have a specific stronger adsorption capacity for laminin compared with other proteins [16], and the mechanisms governing such protein–polymer interactions include polyelectrolyte absorption and hydrophobic and ionic interactions [17]. Therefore, we assumed that PDLLA and LN332 might be deposited layer by layer on Ti through an LBL-like mechanism to achieve enhanced loading amount, controlled release and protein activity maintenance.

As one of the key events in soft tissue sealing establishment, JE formation and adhesion to the implant surface may not only come from the proliferation and migration of nearby mature keratinocytes to the implant surface but also arise from the differentiation of stem cells to keratinocytes. It has been found that stem cell populations exist around the JE to maintain the homeostasis and repair of JE [18]. Mesenchymal stem cells derived from gingival lamina propria (GMSCs) have been proved to own strong wound healing abilities and regenerative potential, and transdifferentiation of human GMSCs into functional keratinocytes has been observed [19, 20]. In addition, Atsuta *et al.* have found that the application of stem cells could enhance the soft tissue sealing around the oral implants [21]. According to the above-mentioned, we speculated that GMSCs might also play a crucial role in the formation of peri-implant epithelial sealing via transdifferentiation into keratinocytes.

This study intended to construct a PDLLA-LN332 composite coating on Ti by an LBL-like method. Its effects on the functions of keratinocytes and the epithelial transdifferentiation of GMSCs were studied, providing a basis for further study to improve the peri-implant soft tissue sealing.

## Materials and methods

### Sample preparation

Commercially pure Ti disks (15 mm in diameter and 1.5 mm in thickness) of grade 1 were provided by Baoji Titanium Industry Co. Ltd, Shaanxi, China. First, the commercially pure Ti disks were polished with sandpaper (400, 800, 1200, 1500, 2000 and 3000 meshes) and cleaned sequentially in acetone, ethanol and deionized water to generate the polished Ti surface (Ti group). Second, 100 mg of PDLLA (Sigma-Aldrich, USA) was dissolved in 10 ml of ethyl acetate, and the formed PDLLA solution was added dropwise on the polished Ti disks to generate the PDLLA surface (TiP group) after the organic solvents were volatilized. The PDLLA-coated samples were covered with 100 μg/ml of LN332 protein solution (BioLamina, USA) and kept for 24 h at 4°C. Afterward, the samples were put into a vacuum freeze dryer (SIM, USA) to remove the water. The samples were then sequentially deposited with the PDLLA solution and the LN332 solution twice to generate the PDLLA-LN332 coating (TiPLN group).

### Sample characterization

Field-emission scanning electron microscopy (FE-SEM, Hitachi S-4800, Japan) and an atomic force microscope (AFM, Shimadzu, Japan) were utilized to observe the surface morphologies and microstructures of the samples. The surface compositions of the samples were detected by an X-ray photoelectron spectroscopy (XPS, Thermo ESCALAB 250Xi, USA) with an Al Kα (30.0 eV) source. Infrared spectra were recorded by a Fourier transform infrared (FTIR) spectrophotometer (Shimadzu, Japan). Surface wettability was determined with a drop shape analyzer (Kruss, Germany), and deionized water was used as the test liquid.

### Ln332 release

The TiPLN samples were immersed in 2-ml DMSO (Sigma-Aldrich, USA), and after sufficient dissolvement, the supernatant was transferred to a new centrifuge tube to measure the total loading amount of LN332. The TiPLN samples were immersed in phosphate buffer solution (PBS) of 5 ml for 6 h and 1, 3, 5, 7, 9, 11, 14, 21 and 28 days at 37°C to measure the LN332 release. At the predetermined time points, 500 μl of the solution was taken out for study, and the PBS solution with samples immersed in it was replenished. The amounts of released LN332 and the total loading amount were detected by human LAMA3 ELISA kits (Cusabio, China). The percentage of protein release was calculated by dividing the accumulated amount of released protein by the total loading amount.

### Effects of the coatings on human keratinocyte functions

#### HaCaT cell culture

HaCaT cells, as human immortalized keratinocytes, were purchased from Shanghai Zhong Qiao Xin Zhou Biotechnology, which were cultured in Dulbecco’s modified Eagle’s medium (DMEM, Gibco, USA) supplemented with 10% fetal bovine serum (FBS, PCM, China) and 1% penicillin/streptomycin (Gibco, USA). The cells were incubated at 37°C in a constant humidity incubator with 5% CO_2_. Every 2–3 days, the fresh culture medium was changed.

#### Cell morphology and cytoskeleton observation

To observe the HaCaT cell morphology on the coatings, 1-ml cell suspension of 2 × 10^4^/ml was seeded on the samples placed in 24-well plates. The unattached cells were removed by rinsing the cells thrice with PBS after 6, 12 and 24 h of incubation. Cells were fixed with 2.5% glutaraldehyde (GA) at 4°C overnight and dehydrated with graded ethanol (20, 40, 60, 80, 90, 95 and 100 vol%). After drying with hexamethyl disilylamine (Macklin, China), the cells were sputter-coated with gold and observed by FE-SEM.

For the cytoskeleton staining, after 2, 6 and 24 h of incubation, the cells were washed thrice with PBS, fixed with 4% paraformaldehyde (PFA) for 20 min and permeabilized by 0.5% TritonX-100 for 5 min. Afterward, the cells were stained with Acti-stain™ 488 phalloidin (Cytoskeleton, USA) for 30 min and counterstained with 4',6-diamidino-2-phenylindole (DAPI) in the dark. The cytoskeleton was then observed with a confocal laser-scanning microscope (CLSM, Nikon, Japan).

#### Cellular adhesion

The cell seeding procedure is the same as in Cell morphology and cytoskeleton observation section. The DAPI staining was performed on different samples to study the early cellular adhesion. At 6 and 24 h, the cells were washed with PBS, fixed with 4% PFA for 20 min, permeabilized with 0.5% Triton X-100 for 5 min and finally, stained with DAPI for 5 min. CLSM was used to observe and acquire the fluorescence photographs.

#### Cell migration

A wound healing assay was performed to evaluate the cell migration ability. After 1-ml cell suspension of 5 × 10^4^/ml being plated on samples and cultured for 2 days to reach confluence, wounds of the cell monolayers were generated with a pipette tip. After another 12 and 24 h of culture in fresh DMEM with 2% FBS, the samples were rinsed with PBS thrice and fixed with 4% PFA for 20 min. The method of cytoskeleton staining is the same as in Cell morphology and cytoskeleton observation section. The fluorescence photographs of wounding regions were obtained with CLSM. The cell migration rate was calculated by the following formula: Relative Migration Rate (%) = (R0—Rn)/R0 × 100%, where R0 represents the initial scratch area and Rn represents the remaining unclosed scratch area.

#### Cell proliferation

Cell proliferation was examined using a Cell Counting Kit-8 (CCK8, Dojindo, Japan). Specifically, the samples were rinsed with PBS thrice at prescribed culturing time points (1, 3, 5 and 7 days). Afterward, a fresh medium of 0.5 ml containing 10% CCK8 was added to each sample and incubated for another 2 h. Then, the optical density of the cell culture medium (100 μl) was detected using a microplate reader at 450 nm (BioTeK, USA).

#### Protein expression

HD-related proteins were assayed by immunofluorescence and western blot. For immunofluorescence, the HaCaT cells were seeded on different samples in a 24-well plate at an initial density of 2 × 10^4^ cells/well. After 48 h of incubation, the cells were washed thrice with PBS, fixed with 4% PFA for 20 min and permeabilized by 0.5% TritonX-100 for 20 min. After washing with PBS, unspecific binding sites were blocked with 10% goat serum for 60 min. Then, the cells were incubated with specific primary antibody against integrin α6, integrin β4, LN332 and plectin at 4°C overnight. The cells were further treated with fluorochrome-conjugated secondary antibody for 1 h and counterstained with DAPI in the dark, and images of the staining were taken with CLSM.

For western blot, the HaCaT cells were seeded on different samples in a 24-well plate at an initial density of 5 × 10^4^ cells/well and incubated for 3 and 7 days. The cells were lysed in RIPA lysis buffer (Beyotime, China), and proteins were harvested. The protein concentrations were measured with a BCA kit (Beyotime, China). Equalized amounts of protein extracts were mixed with loading buffer, boiled for 10 min at 100°C, separated by sodium dodecyl sulfate-polyacrylamide gel electrophoresis, and transferred to polyvinylidene difluoride membranes. After blocking the non-specific adsorption sites with blocking solution (NCM, China), the membranes were incubated with specific primary antibody against integrin α6, integrin β4, laminin332, plectin, β-actin, phosphatidylinositol 3-kinase (PI3K), p-PI3K, serine/threonine protein kinase B (Akt), p-Akt and glyceraldehyde-3-phosphate dehydrogenase (GAPDH) at 4°C overnight. The membranes were further treated with peroxidase-conjugated secondary antibody for 1 h. Eventually, the detection was performed with enhanced chemiluminescence (GeneTex, USA) using a luminescent imager (Bio-Rad, USA). The detailed information on antibodies used is shown in Supplementary Table S1.

#### Integrin β4-PI3K-Akt pathway regulation

The PI3K inhibitor LY294002 (InCellGene, Germany) was used to identify the integrin β4-PI3K-Akt signaling pathway. The HaCaT cells were seeded on TiPLN placed in a 24-well plate with a fresh medium containing 50 μM LY294002 or not. After culturing for 24 h, the integrin β4, PI3K, p-PI3K, Akt, p-Akt and GAPDH were assayed by western blot (refer to Protein expression section for details). The cytoskeleton staining was implemented to observe the cytoskeleton (refer to Cell morphology and cytoskeleton observation section for details), and the DAPI staining was performed to investigate the early cellular adhesion (refer to 2.4.3 for details).

The cells were seeded on the TiPLN surface in a 24-well plate, and wounds of the cell monolayers were made with a pipette tip after reaching confluence. Then, the cells were incubated for another 24 h with LY294002 or not to evaluate the cell migration ability (refer to Cell migration section for details).

The HaCaT cells were seeded on TiPLN placed in a 24-well plate. One group was cultured without LY294002 for all 7 days, whereas the other group was cultured with LY294002 for the first 5 days and without LY294002 for the other 2 days. After a total of 7 days of culturing, the CCK8 assay was used to examine cell proliferation (refer to Cell proliferation section for details).

### Effects of the coatings on GMSC functions and epithelial differentiation

#### Isolation and identification of primary GMSC culture

The human gingival tissues were obtained as remnants of discarded tissues of third molar extractions from healthy human subjects aged 18–25 years who underwent a dental procedure under the approved Institutional Review Board (IRB) protocol at Hospital of Stomatology, the Fourth Military Medical University. After being rinsed thrice with PBS with 2% penicillin/streptomycin, the epithelial layer was removed after incubation in 2.5 mg/ml dispase II (Sigma-Aldrich, USA) solution overnight at 4°C. Then the tissues were minced and digested in 4 mg/ml collagenase IV (Sigma-Aldrich, USA) at 37°C for 2 h. Afterward, the tissues were washed with PBS, resuspended and seeded in a six-well plate with a complete alpha-minimum essential medium (α-MEM, Gibco, USA) supplemented with 10% FBS and 1% penicillin/streptomycin. The cells between passages 3–5 were used for the experiments.

A flow cytometric analysis was used to study the expression of MSC surface markers, including CD44, CD90 and CD105, and hematopoietic cell markers, including CD14, CD34 and CD45, to confirm the MSC characteristics of the cells obtained. All primary antibodies used in the study are shown in Supplementary Table S1. Differentiation induction experiments were also performed to identify the multiple differentiation potential of GMSCs, including adipogenesis and osteogenesis. The culture medium was replaced with an osteogenic induction medium containing 50 nM dexamethasone, 50 μg/ml ascorbic acid (AA) and 10 mM β-glycerophosphate (all from Sigma-Aldrich) when the cells achieved 60–70% confluency to investigate the osteogenic potential. After 1 week of culturing, the cells were fixed with 4% PFA, and alkaline phosphatase (ALP) staining was performed using an ALP kit (LeaGene China). After 3 weeks of culturing, the cells were fixed with 4% PFA and incubated with 2% Alizarin Red S staining (Beyotime, China). The cells were cultured in an adipoinduction medium supplemented with 0.5 mM methylisobutylxanthine, 10 μg/ml hydrocortisone, 1 μM dexamethasone and 10 μM indomethacin (all from Sigma-Aldrich) for 3 weeks for adipogenic differentiation. Then, the cultures were fixed with 4% PFA and stained with fresh Oil Red O solution (LeaGene, China).

#### Cell morphology, cytoskeleton observation, adhesion and proliferation

One milliliter cell suspension of 2 × 10^4^ cells/ml was seeded on different samples in 24-well plates. After culturing for 6, 12 and 24 h, the cells were fixed with 2.5% GA, dehydrated with graded ethanol, critical point drying and gold spraying. After culturing for 2, 6 and 24 h, fluorescein isothiocyanate phalloidin and DAPI were added to stain F-actin and nucleus, respectively. The FE-SEM was used to observe the cellular morphology, and the CLSM was utilized to observe the cytoskeleton staining (refer to Cell morphology and cytoskeleton observation section for details). After culturing for 6 and 24 h, the DAPI staining was performed, and the CLSM was used to observe and acquire the fluorescence photographs (refer to Cellular adhesion section for details). After culturing for 1, 3, 5 and 7 days, the CCK8 assay was performed (refer to Cell proliferation section for details).

#### Epithelial differentiation of GMSCs

To evaluate the epithelial differentiation potential, 1 ml GMSC suspension of 2 × 10^4^ cells/ml was cultured on different samples with αMEM and 10% FBS to reach 60–70% confluence. Then, the epithelial induction supplements (EIS) consisting of 1 μM all-trans retinoic acid (ATRA, Sigma-Aldrich, USA), 25 ng/ml bone morphogenetic protein 4 (BMP4, Peprotech, USA), 20 ng/ml epidermal growth factor (EGF, Peprotech, USA) and 0.3 mM AA were added, and the serum concentration decreased to 2%. Different culture mediums of mere αMEM and mixtures of keratinocyte serum-free medium (Gibco, USA) and αMEM with ratios of 1:3, 1:1 and 3:1 were sequentially used for a 2-day duration. After a total of 7 days of culturing in the EIS-containing medium, the western blot was used to detect the expression of cytokeratin 14 (CK14), CK18, CK19 and β-actin.

### Statistical analysis

All data are expressed as mean ± standard deviation. GraphPad Prism 8.0 software package was used for statistical analysis. Experimental data were compared using one-way ANOVA and Tukey’s multiple comparison test or two-tailed Student’s *t*-test, and the value of *P *<* *0.05 was considered statistically significant. Experiments were performed at least three times to ensure consistent results.

## Results and discussion

### Surface characterization

**Figure 1.**
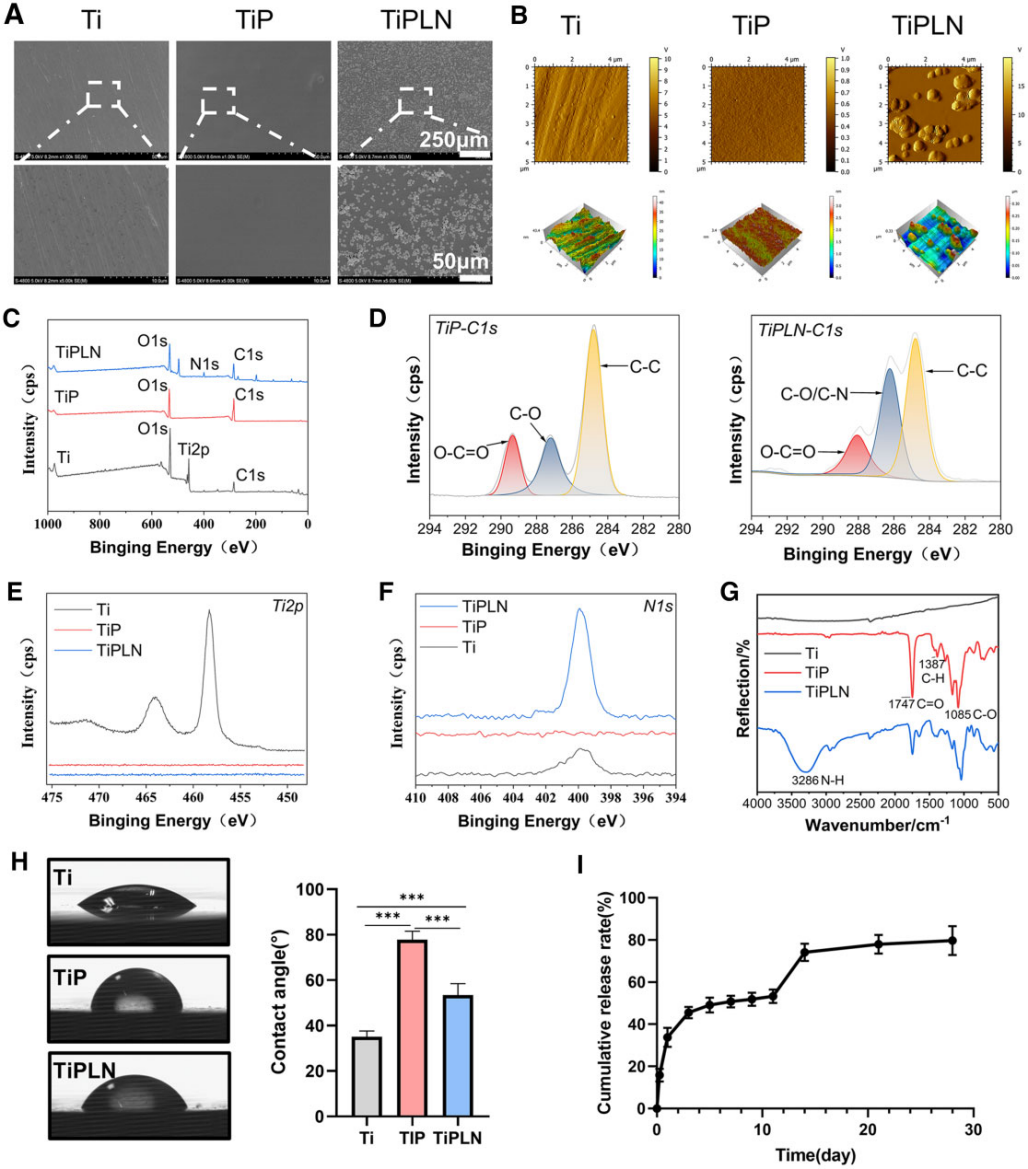
Implant surface characterizations of different Ti plates. (**A**) FE-SEM observation of Ti, TiP and TiPLN. (**B**) Phase contrast and 3D of surface height fluctuations of AFM scanning images (5 μm × 5 μm) of Ti, TiP and TiPLN. (**C**) XPS survey spectra of Ti, TiP and TiPLN samples. (**D**) The peak fitting of high-resolution C1s for TiP and TiPLN surfaces. (**E**) High-resolution T2p for Ti, TiP and TiPLN substrates. (**F**) High-resolution N1s for Ti, TiP and TiPLN substrates. (**G**) FTIR spectra of Ti, TiP and TiPLN substrates. (**H**) Water contact angle measurement of Ti, TiP and TiPLN. (**I**) The cumulative release rate of preloaded LN332 from TiPLN in PBS (pH 7.4) at 37°C for 28 days (***P <* *0.001).

FE-SEM was used to observe the surface morphologies of Ti, TiP and TiPLN (Fig. 1A). Ti exhibited some artificial scratches due to the polishing process, whereas TiP showed an extremely smooth surface. TiPLN exhibited abundant globular particles on its surface, which have well-defined 3D smooth globular contours and should be the LN332 proteins. The sizes of the globular particles were around 2–3 μm, suggesting the formation of oligomers since the size of an extended LN332 molecule is 120 nm at most [22]. Tapia-Lopez *et al.* have used LN332 protein to modify zirconia and found that the sizes of LN332 protein are around 200–400 nm with a little squashed shape [12]. The variance in the shape and size of LN332 after loading onto biomaterials may be associated with the different biomaterial substrates, protein concentration and binding mechanism.

AFM can reflect not only biomaterial topography but also surface roughness. The AFM topographic images of different samples are shown in Fig. 1B. The topography is well aligned with the SEM views in Fig. 1A. The Ra and Rq values of Ti, TiP and TiPLN are 4.52 ± 0.54 nm/5.83 ± 0.69 nm, 0.41 ± 0.14 nm/0.52 ± 0.18 nm and 39.00 ± 4.68 nm/53.83 ± 8.91 nm, respectively. We can see that the PDLLA film formation reduces the surface roughness of Ti from 4.52 to 0.41 nm because the PDLLA film can expunge irregular topography on the Ti substrate. The higher surface roughness of TiPLN is due to the presence of protein particles. According to the average roughness, the implant surfaces can be divided into four grades: smooth (<0.5 μm), minimum roughness (0.5–1 μm), medium roughness (1–2 μm) and high roughness (>2 μm) [23]. Although the morphology and roughness of the samples change significantly after PDLLA and LN332 loading, their Ra values are <0.1 μm and belong to the category of smooth surface, which should have minimal influence on cell behavior [24].

The hydrophilicity of the samples was analyzed by contact angle measurement, and the results are shown in Fig. 1H. The water contact angle of the Ti is about 35.06°, indicating its hydrophilic nature. After depositing the PDLLA, the contact angle increased up to 77.77°, which is consistent with the literature (71.5°) [16]. Compared with TiP, TiPLN possesses a reduced contact angle of 53.42°, which is still larger than that of Ti. LN332 has both hydrophobic and hydrophilic sites [12]. After the binding of hydrophobic sites with PDLLA, the hydrophilic sites are accordingly exposed, leading to enhanced hydrophilicity.

The elemental composition and binding energies were monitored by XPS. Although Ti, carbon (C), nitrogen (N) and oxygen (O) were detectable on all samples, the obvious difference in peak strength indicated that the composition of the sample surfaces changed (Fig. 1C–F). C is the main element of PDLLA; thus, there was a clear increase in the elemental content of C on the TiP and TiPLN substrates compared with the polished Ti (Fig. 1C). The Ti signals on TiP and TiPLN disappeared (Fig. 1E), confirming that for TiP and TiPLN, the Ti substrates have been fully covered with PDLLA. The C1s of TiP had three resolved peaks at 284.80, 287.25 and 289.35 eV (Fig. 1D and Table 1), corresponding to the following functional groups: C–C (55.77%), C–O (25.27%) and O=C–O (18.66%) [25, 26]. For TiPLN with LN332 loading, the three resolved peaks were still retained but shifted and changed in the ratio, with the peaks at 284.80, 286.25 and 288.05 eV corresponding to C–C (47.33%), C–O/C–N (36.44%) and O = C–O (16.23%). The presence of C–N in LN332 should have led to the increase in the ratio of C–O/C–N and a certain shift in the peaks. As shown in Fig. 1F, the nitrogen content of 1.14% was observed on the polished Ti surface, which was probably related to exposure to air before XPS analysis [27]. No nitrogen was observed on TiP. An N1s peak was detected at 399.9 eV on TiPLN, and the nitrogen content was up to 7.38% because of the N content in the amine groups of protein [10].

**Table 1.** Surface elemental percentages of C, N, O, Ti of Ti, TiP and TiPLN, and C1s peaks percent relative areas of TiP and TiPLN

Samples	Elemental percentage (%)				C1s relative area (%)		
	C	N	O	Ti	O=C–O	C–O/C–N	C–C
Ti	10.32 ± 0.02	1.14 ± 0.24	53.19 ± 1.90	35.16 ± 1.69			
TiP	47.92 ± 0.13	0	52.08 ± 0.13	0	18.66	25.57	55.77
TiPLN	32.30 ± 0.02	7.38 ± 0.41	60.32 ± 0.41	0	16.23	36.44	47.33

The surface chemical structures of the samples were analyzed by FTIR spectroscopy (Fig. 1G). Ti did not display any absorption peak. For TiP, the strong peak at around 1747 cm^−1^ was assigned to the stretching vibration of C=O, that at 1387 cm^−1^ was attributed to the C–H bending and that at 1085 cm^−1^ was attributed to the C–O stretching [27, 28]. For TiPLN, the absorption peak at 3286 cm^−1^ was ascribed to N–H stretching [29].

In summary, all the above-described results clearly demonstrate that the PDLLA coating and the PDLLA-LN332 composite coating can be evenly formed on the Ti substrate.

### Ln332 release from TiPLN

After the TiPLN samples are immersed in PBS, LN332 on the top layer will diffuse and dissolve first, and then the PDLLA will gradually degrade and release the underlying LN332. Therefore, LN332 will be gradually released with the extension of immersion time, and the PDLLA acts as a controlled releasing platform. As shown in Fig. 1I, ∼34% of the initial loading amount was released from TiPLN on Day 1, 16% was released during Days 1–5, 4% during Days 5–11, 21% was released during Days 11–14 and 5% was released during Days 14–28. Hence, the LN332 proteins are released at staged rates with time. The first LN332 burst release during Days 0–5 was ascribed to the diffusion and subsequent dissolution of LN332 adsorbed on the top-layered PDLLA to the surrounding solution [30]. The second burst release during Days 11–14 was ascribed to the diffusion of the lower-layered LN332 into the solution with the degradation of PDLLA [30]. The epithelial cells of the peri-implant mucosa migrate and proliferate to establish initial peri-implant epithelial sealing within 2 weeks after implantation, and the epithelial sealing is usually formed within 4 weeks [31]. Hence, the staged LN332 release lasting as long as 28 days from the PDLLA-LN332 coating is expected to achieve a more satisfactory peri-implant epithelial sealing.

### The HaCaT cell morphology, adhesion, migration and proliferation

**Figure 2.**
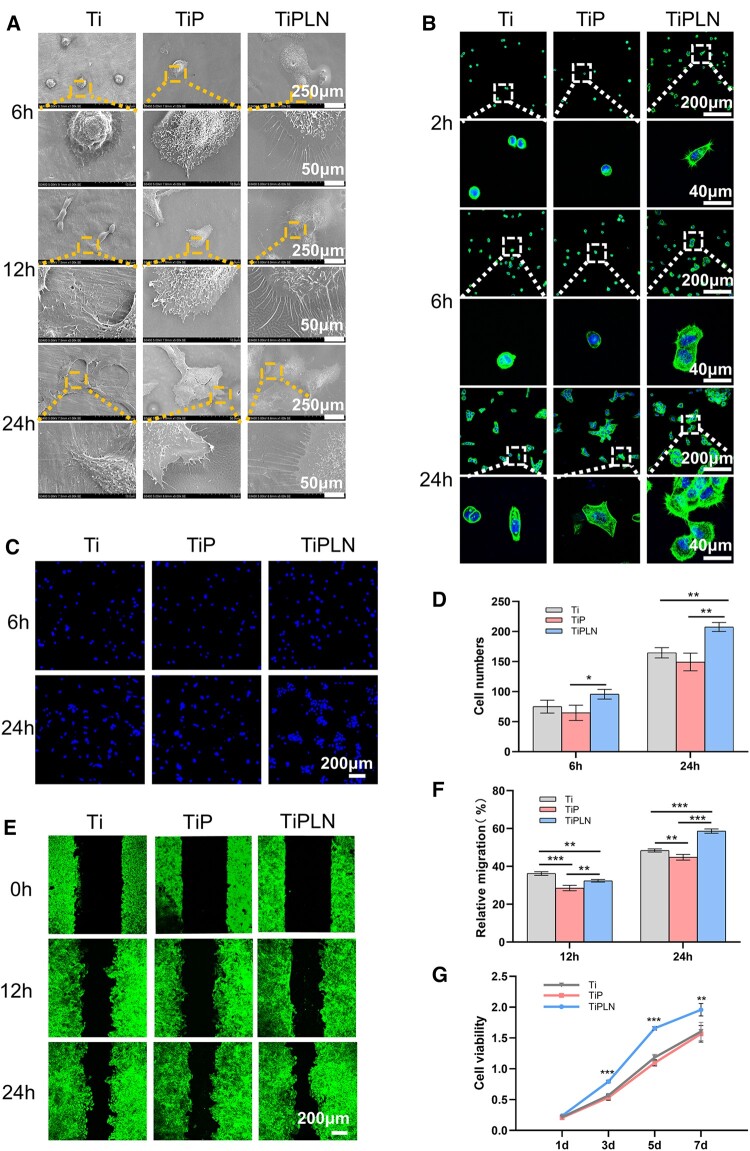
The morphology, adhesion, migration and proliferation of HaCaT cells. (**A**) SEM morphology of HaCaT cells on different substrates for 6, 12 and 24 h; (**B**) CLSM images of the stained cytoskeleton of HaCaT cells on different substrates for 2, 6 and 24 h. (**C**) Representative nuclei staining (**D**) and quantitative counting of adherent cells on different substrates for 6 and 24 h incubation. (**E**) CLSM images of the stained cytoskeleton (**F**) and relative migration rate of HaCaT cells on different substrates in the scratch-wound healing assay. (**G**) Cell growth curves of CCK8 assay for HaCaT cells on different substrates (*P <* *0.05; **P <* *0.01; ***P <* *0.001).

Improving the attachment between the epithelial cells and the Ti surface can lead to augmented soft tissue sealing and, thus, reduced the risk of infections. We performed the SEM observation after 6, 12 and 24 h of culturing and the fluorescence staining after 2, 6 and 24 h of culturing to observe the initial cellular spreading behavior on various substrates. On TiPLN, the HaCaT cells exhibited significantly quicker cell colonization and spreading as early as 2 h after seeding, much larger cell spreading area at all time points, and more and longer filopodia or better actin cytoskeleton extension compared to those on Ti and TiP (Fig. 2A and B). As shown in Fig. 2C and D, the initial number of adherent cells on the different substrates increased from 6 to 24 h. TiPLN induced more adherent cells than TiP surfaces at 6 h (*P < *0.05) and significantly more ones than Ti and TiP at 24 h (*P < *0.01). More cells were observed on TiPLN than on Ti at 6 h, although there was no statistical difference. The results indicate that TiPLN not only facilitates keratinocyte early colonization and spreading but also benefits keratinocyte adhesion.

LN332 is an important cellular adhesion molecule. It participates in the establishment of HDs and binds to ECM through integrin α6β4 to promote cellular adhesion [32]. Tamura *et al*. [9] have constructed LN332 coating by immersing Ti alloy into LN332 protein solution and found that the adhesion ability of gingival epithelial cells was enhanced. Tapia-Lopez *et al.* [12] have etched and activated the zirconia surface with argon plasma, and then LN332 was bonded on the zirconia surface by adsorption, which significantly improved epithelial cell spreading and adhesion. Our results are consistent with theirs, collectively confirming the enhancing effect of LN332 on epithelial cell/keratinocyte spreading and adhesion.

The epithelial cell migration is an essential process in soft tissue sealing following implantation. The scratch-wound healing assay was performed to evaluate the migration ability of HaCaT cells. The fluorescence staining images and quantification of wound healing rates are shown in Fig. 2E and F. The migration ability of HaCaT cells on TiP was the poorest, and this should be associated with its lowest hydrophilicity and lack of an active group. The migration ability of HaCaT cells on TiPLN is poorer at 12 h but better at 24 h than that on Ti. Notably, during the wounding of the cell monolayers, the culture medium was refreshed. At 12 h, there was a limited amount of LN332 released; hence, the cell migration might be mainly affected by surface properties, such as hydrophilicity. Due to the less hydrophilic nature of TiPLN and TiP, they impair the migration ability of HaCaT cells. Up to 24 h, enough amount of LN332 was released from TiPLN to show promoting effect on the cell migration.

The proliferative activity of the HaCaT cells on different samples after 1, 3, 5 and 7 days was detected (Fig. 2G). The cell proliferation on all the samples showed an upward trend with time. Except for the first day, the cell proliferative activity on TiPLN was significantly higher than that on Ti and TiP (*P *<* *0.01), suggesting the enhancing effect of TiPLN on the keratinocyte proliferation.

To conclude, the PDLLA-LN332 composite coating containing LN332, the main component of the BM, is demonstrated to be effective in promoting the adhesion, spreading, migration and proliferation of keratinocytes. In line with our results, functionalization of Ti implant with LN332-derived peptide and LN332 gene was found to promote the adhesion, migration and proliferation of epithelial cells [4, 9, 32, 33]. Hence, TiPLN can give rise to high-quality PIE attachment and, thus, good soft tissue sealing.

### Western blot analysis of HD-related proteins

HDs are known as specific cellular adhesion structures facilitating the adhesion between the enamel of the tooth and junctional epithelial cells and also between the Ti implant and peri-implant epithelial cells [3, 8]. LN332, integrin a6β4 and plectin are important constituents of HDs. Particularly, LN332 connects with HDs directly and enhances HD assembly by keratinocytes as a principal constituent of the BM. Moreover, LN332 is deposited into the wound bed by keratinocytes at the leading edge of the epithelium after an injury [7]. The deposited LN332 repairs the BM and re-establishes the epithelial anchorage to the BM via integrin α6β4 in HDs [6, 34]. After implantation surgery, the soft tissue sealing is accompanied by LN332 and integrin α6β4-mediated keratinocyte adhesion, migration, proliferation and PIE attachment to the implant surface via the formation of HDs [33, 34]. Besides forming mechanical anchorage with LN332, integrin α6β4 is also an important transmembrane receptor to mediate the bidirectional flow of information from ECM to cells and regulate the cellular adhesion, migration and proliferation [35]. Plectin interacts with integrin β4 and the intracellular actin filaments to associate ECM with the cytoskeleton, providing mechanical support for epithelial cells and influencing cellular adhesion and migration [36].

**Figure 3.**
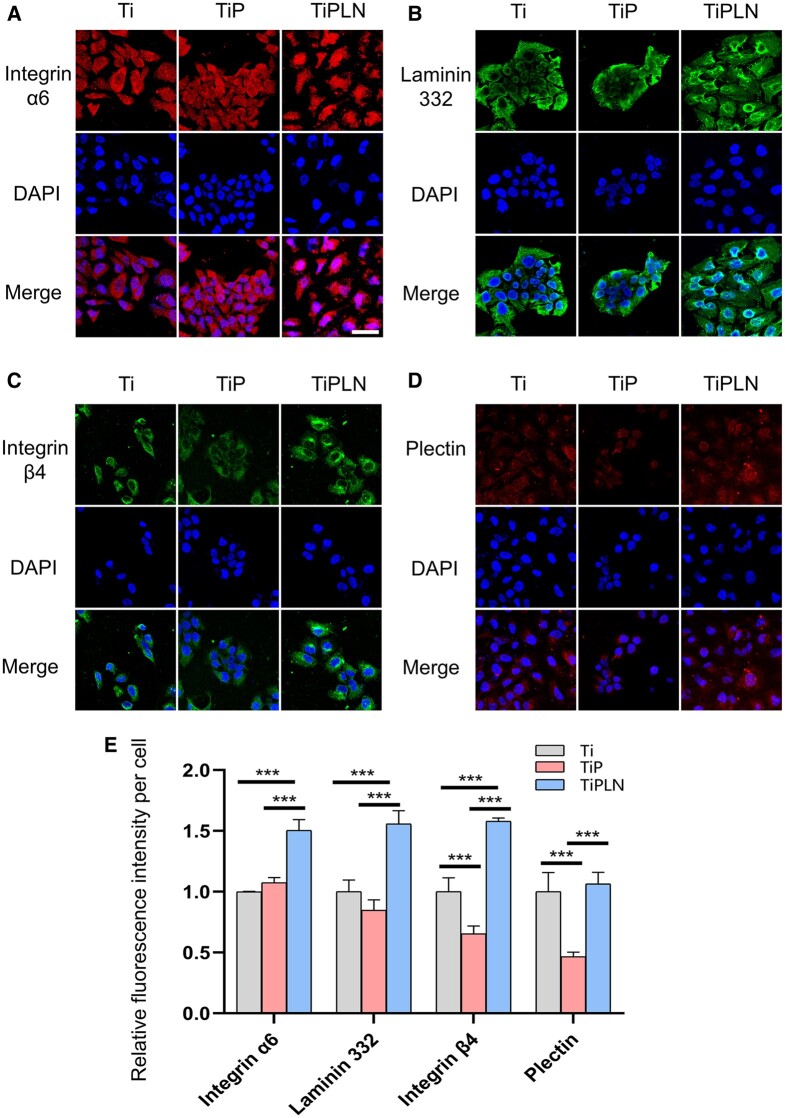
Immunofluorescence staining and semi-quantification. Immunofluorescence staining for (**A**) integrin α6, (**B**) LN332, (**C**) integrin β4 and (**D**) plectin in HaCaT cells cultured for 48 h on different substrates. (**E**) Relative fluorescence intensity per cell of integrin α6, LN332, integrin β4 and plectin of HaCaT cells (***P <* *0.001).

**Figure 4.**
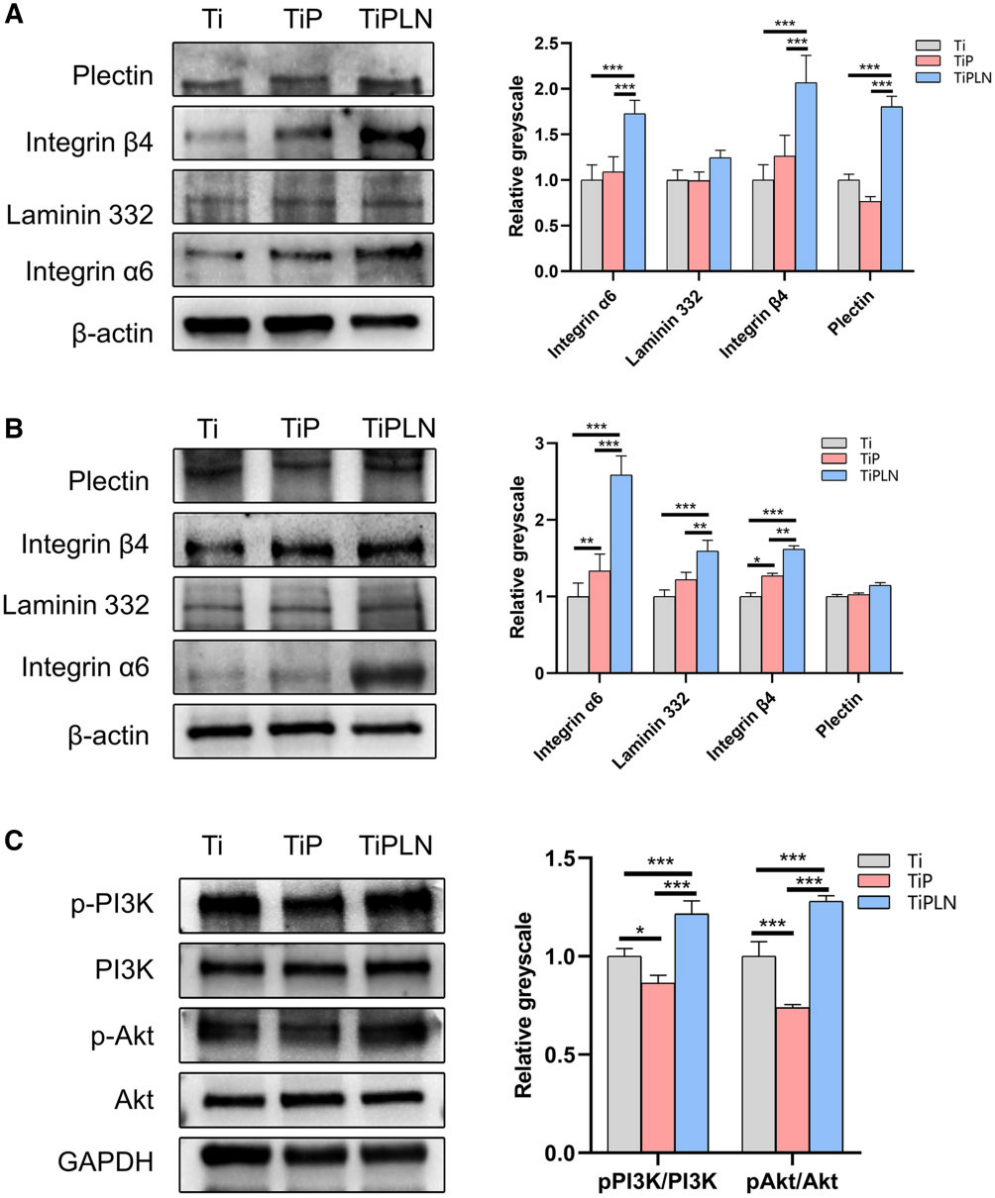
Western blot analysis and semi-quantification. (**A**) Western blot analysis and semi-quantification of integrin α6, laminin332, integrin β4 and plectin of HaCaT cells on different substrates at 3 days. (**B**) Western blot analysis and semi-quantification of integrin α6, laminin332, integrin β4 and plectin of HaCaT cells on different substrates at 7 days. (**C**) Western blot analysis and semi-quantification of pPI3K, PI3K, pAkt and Akt of HaCaT cells of different substrates (*P <* *0.05; **P <* *0.01; ***P <* *0.001).

Immunofluorescence analysis (Fig. 3A–D) showed that the HD-related proteins integrin α6 (red), integrin β4 (green), LN332 (green) and plectin (red) all distributed in the cytoplasm. The expression levels of integrin α6, LN332 and integrin β4 (Fig. 3A–C) by the cells on TiPLN were significantly higher than those on TiP and Ti. The expression of plectin (Fig. 3D) on Ti and TiPLN was higher than that on TiP. The protein levels of integrin α6β4, LN332 and plectin at 3 and 7 days were detected using western blot, and the results are shown in Fig. 4A and B. On Day 3, there was no significant difference in the LN332 expression among the samples. However, on Day 7, the LN332 expression on TiPLN was significantly higher than on TiP and Ti, indicating that TiPLN coating can further promote the LN332 expression by keratinocytes. Our results showed that the expressions of integrin α6 and β4 on TiPLN were significantly higher than those on TiP and Ti. The expression of plectin on TiPLN was higher than that of TiP and Ti on Day 3, while no significant difference was found on Day 7. The PDLLA-LN332 composite coating can improve the protein expression levels of integrin α6β4, LN332 and plectin. Ghorbani *et al*. [37] have studied the role of HDs by protein nanopatterning and found the pattern of LN332 can determine the cellular selection of adhesion types with a size-dependent initiation and maturation of HDs. The immobilization of LN332-derived peptide [4, 33] and loading of laminin332 gene [32] on the Ti surface have been reported to promote the formation of HDs and the expression of HD-related proteins, such as integrin α6β4, LN332, plectin and collagen XVII, which is in accordance with our results. The improved expression of integrin α6β4, LN332 and plectin should account for the enhanced adhesion, spreading, migration and proliferation of keratinocytes.

### Integrin β4-PI3K-Akt pathway regulation

Integrin α6β4 can regulate cellular adhesion, cell cycle and cell migration through the PI3K-Akt pathway [38]. The integrin β4 subunit has a unique cytoplasmic domain linked to the actin filament system, which is a crucial determinant for activing PI3K and other signaling pathways [38, 39]. Bon *et al.*’s [40] results have shown that the activity of PI3K and Akt was obviously reduced due to the loss of integrin β4. LN332 can interact with integrin α6β4 and epidermal growth factor receptor to activate the PI3K signaling pathway, providing an autocrine positive feedback loop and contributing to cellular adhesion, migration and invasion [41, 42]. The PI3K, p-PI3K, Akt and p-Akt protein levels were evaluated using the western blot assay to directly observe the effect of the PDLLA-LN332 composite coating on the PI3K-Akt pathway. The p-PI3K and p-Akt levels were greater on TiPLN than that on Ti and TiP (Fig. 4C). The results confirmed that TiPLN could activate the PI3K-Akt pathway, possibly through integrin β4. The chimeric peptide of LN332 immobilized on the Ti surface activated the PI3K-Akt pathway through integrin α6β4, which is consistent with our results [33].

**Figure 5.**
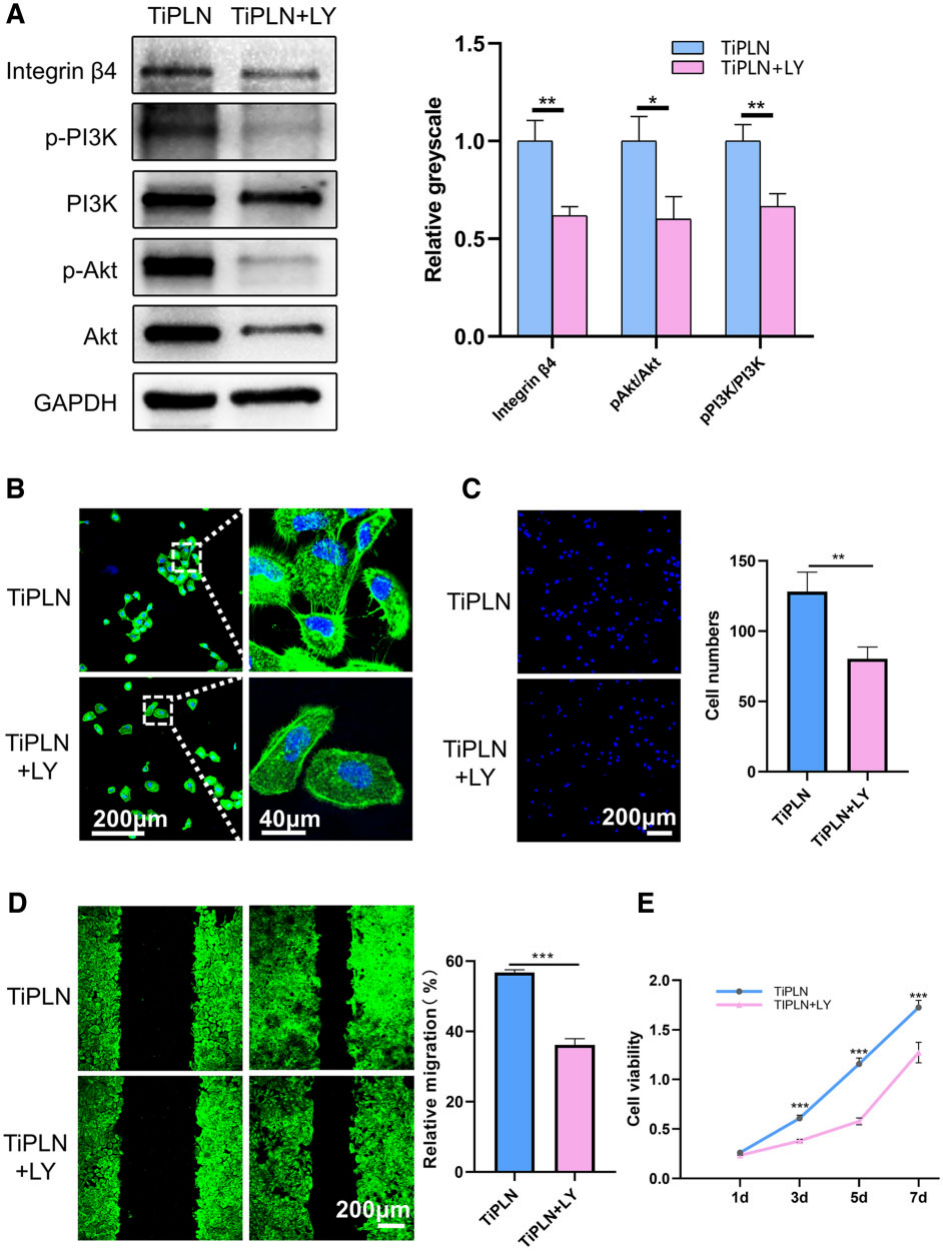
Inhibition of the PI3K pathway (PI3K inhibitor LY294002, 50 μM). (**A**) Western blot analysis and semi-quantification of pPI3K, pAkt and integrin β4 of HaCaT cells on TiPLN and TiPLN+LY. (**B**) CLSM images of the stained cytoskeleton of HaCaT cells on TiPLN and TiPLN+LY at 24 h. (**C**) Representative nuclei staining and quantitative counting of adherent cells on TiPLN and TiPLN+LY at 24 h. (**D**) CLSM images of the stained cytoskeleton and relative migration rate of HaCaT cells on TiPLN and TiPLN+LY in the scratch-wound healing assay at 24 h. (**E**) Cell growth curves of CCK8 assays on TiPLN and TiPLN+LY (*P <* *0.05; **P <* *0.01; ***P <* *0.001).

To further verify this pathway, LY294002 was used to block the PI3K signaling. Then, the cell morphology, adhesion, proliferation, migration and the protein levels of integrin β4, PI3K, p-PI3K, Akt and p-Akt on the PDLLA-LN332 composite coating were inspected, respectively. As shown in Fig. 5A, the expression of p-PI3K and p-Akt was significantly decreased by LY294002, indicating a successful inhibition of this pathway. Interestingly, the expression of integrin β4 was also downregulated by LY294002, possibly due to the positive feedback effect of the LN332/integrin α6β4/PI3K-Akt pathway on integrin expression. The PI3K signaling pathway has been shown to play an important role in the expression of integrin β4 and the construction of actin filaments of epithelial cells on the Ti surface [38].

In the presence of LY294002, the cells showed fewer pseudopodia and intercellular interactions (Fig. 5B), a lower adherent cell number at Day 1 (Fig. 5C), poor migratory ability (Fig. 5D) and significantly decreased cell proliferation number (Fig. 5E). The proliferation rate of cells was significantly inhibited in the presence of LY294002 (the first 5 days) but recovered after depriving LY294002 (after Day 5) to an extent similar to the control group without LY294002. LY294002 is a reversible inhibitor for the PI3K pathway. The rapid recovery of cell proliferation after depriving LY294002 further demonstrates the important role of the PI3K-Akt pathway in the proliferation of keratinocytes on TiPLN.

**Figure 6.**
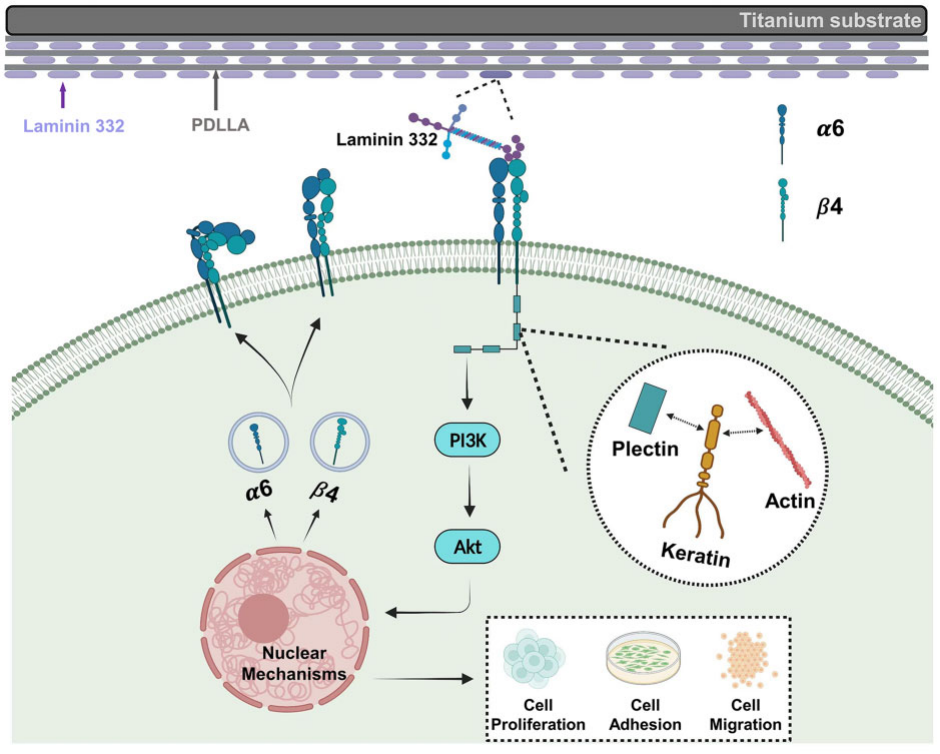
The effects of PDLLA-LN332 composite coating on the behavior of HaCaT cells in terms of the PI3K-Akt signal pathway.

Overall, as shown in Fig. 6, the PDLLA-LN332 composite coating can activate the intracellular PI3K-Akt pathway via binding with the cell integrin α6β4, which can further enhance the expression of integrin α6β4 and promote cellular adhesion, migration and proliferation. Our results are consistent with other studies suggesting that the PI3K pathway activated by integrin α6β4 positively promotes keratinocyte biological behaviors [39].

### GMSC primary cultures and characterization

**Figure 7.**
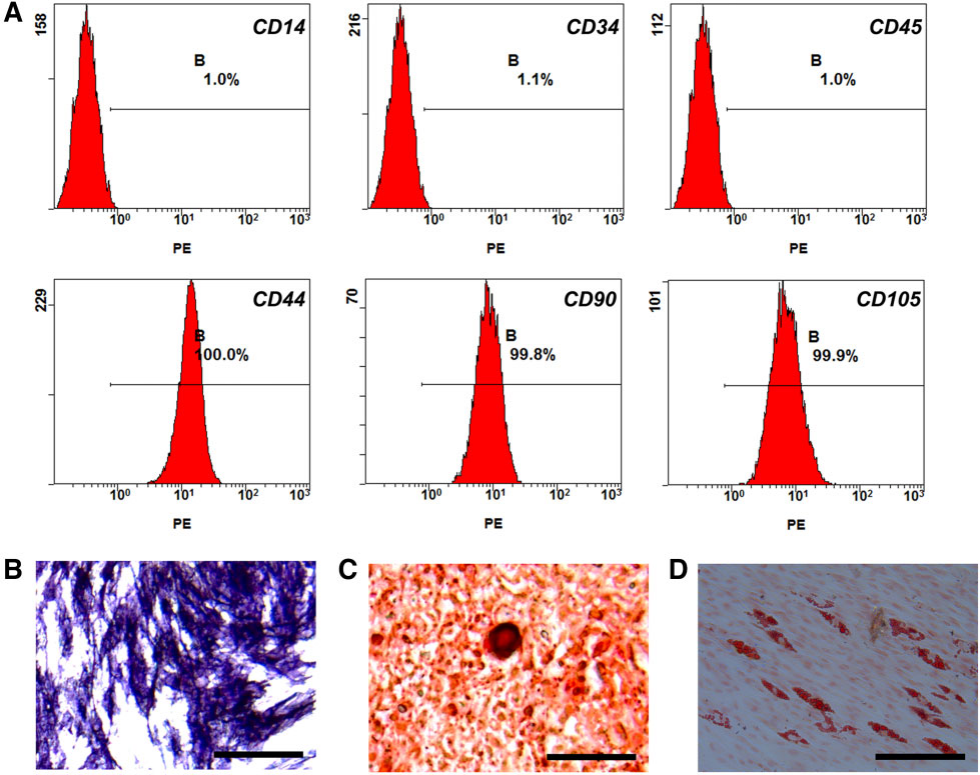
GMSCs characterization. (**A**) Expression of CD14, CD34, CD45, CD44, CD90 and CD105 was assessed by flow cytometry. (**B**) The osteogenesis potential was examined using ALP staining. (**C**) The osteogenesis potential was examined using alizarin red staining. (**D**) The adipogenesis was assessed with oil red O staining (Bar =100 μm).

The flow cytometric analysis outcomes showed that the GMSCs consist of a single phenotypic population positive for CD44, CD90 and CD105. By contrast, the cells were negative for the markers of the hematopoietic lineage, including CD14, CD34 and CD45 (Fig. 7A). The differentiation experiments were performed to identify the multiple differentiation potential of GMSCs. The results showed that the GMSCs could successfully differentiate into osteocytes (Fig. 7B and C) and adipocytes (Fig. 7D).

### GMSC morphology, adhesion and proliferation

**Figure 8.**
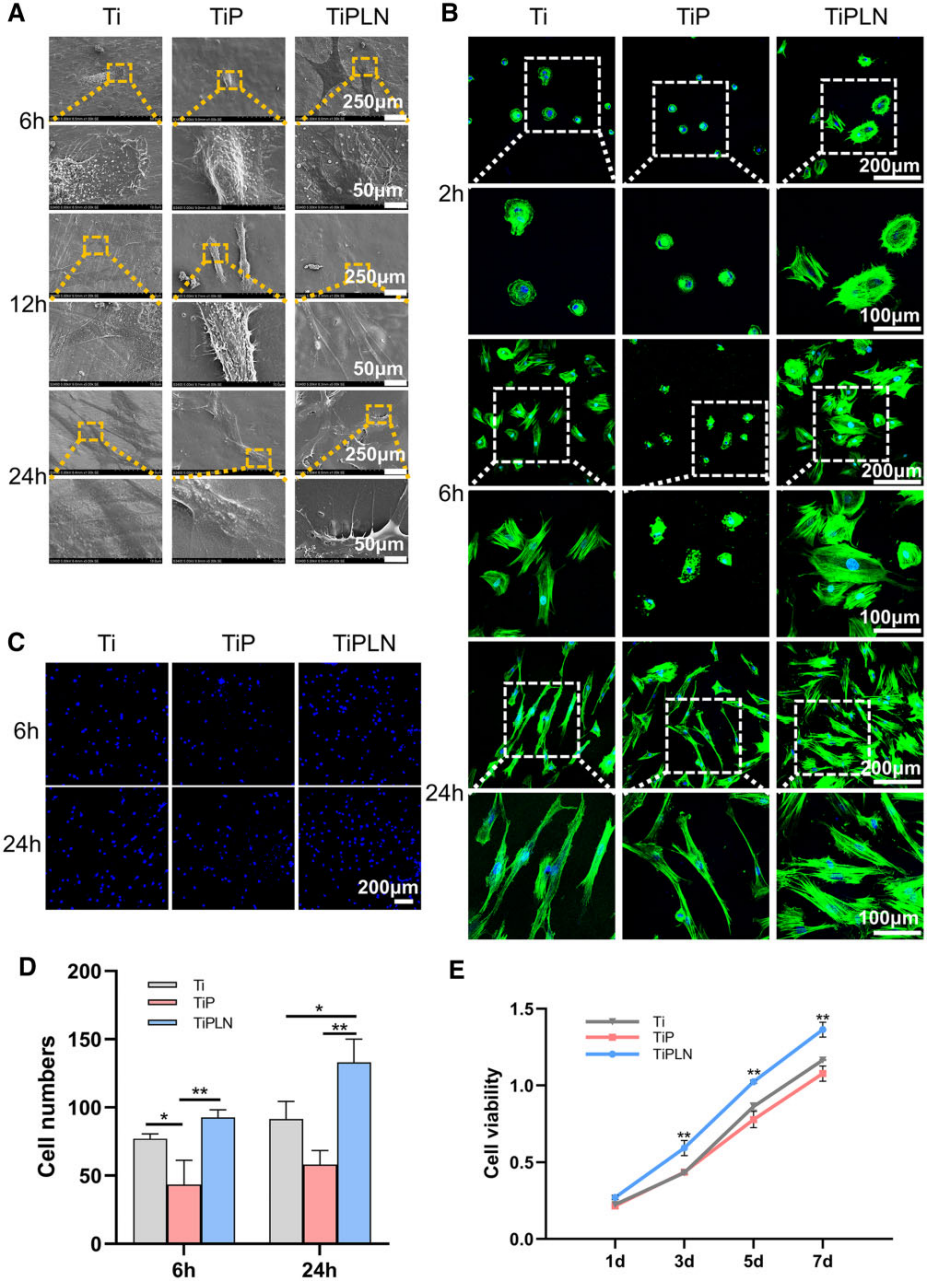
The morphology, adhesion, migration and proliferation of GMSCs. (**A**) SEM morphology of GMSCs cells on different substrates for 6, 12 and 24 h. (**B**) CLSM images of the stained cytoskeleton of GMSCs on different samples for 2, 6 and 24 h. (**C**) Representative staining of nuclei and (**D**) quantitative counting of adherent cells on different substrates after 6 and 24 h of incubation. (**E**) Cell growth curves by CCK8 assays for GMSCs on different substrates (*P <* *0.05; **P <* *0.01).

Given that GMSCs might contribute to the formation of PIE sealing, the effects of the coatings on the morphology, adhesion and proliferation of GMSCs were studied. Figure 8A and B shows the SEM and fluorescent images of GMSCs. GMSCs on TiPLN exhibited better cell spreading, significantly more and longer filopodia or better actin cytoskeleton extension compared to Ti and TiP. The cellular response to surface properties differs depending on cell type, and the physicochemical interactions between cells and substrates prompt the cytoskeleton rearrangement and affect the subsequent cellular functions, including proliferation and differentiation of stem cells [43].

The cellular adhesion on different substrates was investigated by the immunofluorescence staining. As shown in Fig. 8C and D, TiPLN surfaces had significantly more cells than Ti and TiP surfaces at 24 h after culturing (*P < *0.05) and more cells than TiP surfaces at 6 h after culturing (*P < *0.01). As shown in Fig. 8E, the proliferation of cells on different substrates showed an upward trend with time. The cell proliferation among different samples was in the following order except on Day 1: TiPLN > Ti > TiP. TiPLN induced significantly higher proliferation of GMSCs than Ti and TiP (*P *<* *0.01). The results indicate that TiPLN benefits initial adhesion and subsequent proliferation of GMSCs. The addition of LN332 to the culture medium was found to promote the adhesion of bone marrow mesenchymal stem cells [44]. Nonetheless, our study provides the first evidence that LN332 functionalized biomaterial coating could enhance the adhesion, spreading and proliferation of mesenchymal stem cells. TiPLN can improve the behavior of both keratinocytes and GMSCs.

### Epithelial transdifferentiation of GMSCs

Re-epithelialization might be accomplished not only by the proliferation and migration of mature resident cells to the injury site but by the transdifferentiation of stem cells into epithelial cells/keratinocytes [45]. GMSCs exist in the gingival tissue and provide a beneficial effect on wound healing [46]. GMSCs are likely to participate in the healing of peri-implant soft tissue through epithelial transdifferentiation. Cytokines can stimulate specific signaling pathways, which further promote MSC transdifferentiation to a specific cellular lineage by binding to specific cell membrane receptors [47]. ATRA, BMP4, EGF, AA and other factors are conducive to the epithelial differentiation and keratin expression of MSCs [46]. Retinoic acid signaling is a valid inducer of cell differentiation. Brzoska *et al.* [48] have added ATRA to the medium for epithelial differentiation of human adipose-derived stromal cells *in vitro* and increased the expression of CK 18 after 10 days of induction. BMP signaling is one of the dominant pathways participating in keratinocyte differentiation. BMP4, as the key regulator of stem cells among all known BMP family members, induces epithelial differentiation of murine embryonic stem cells [49]. Hence, in this study, ATRA, BMP4, EGF, and AA were used as supplements to induce epithelial differentiation of GMSCs.

**Figure 9.**
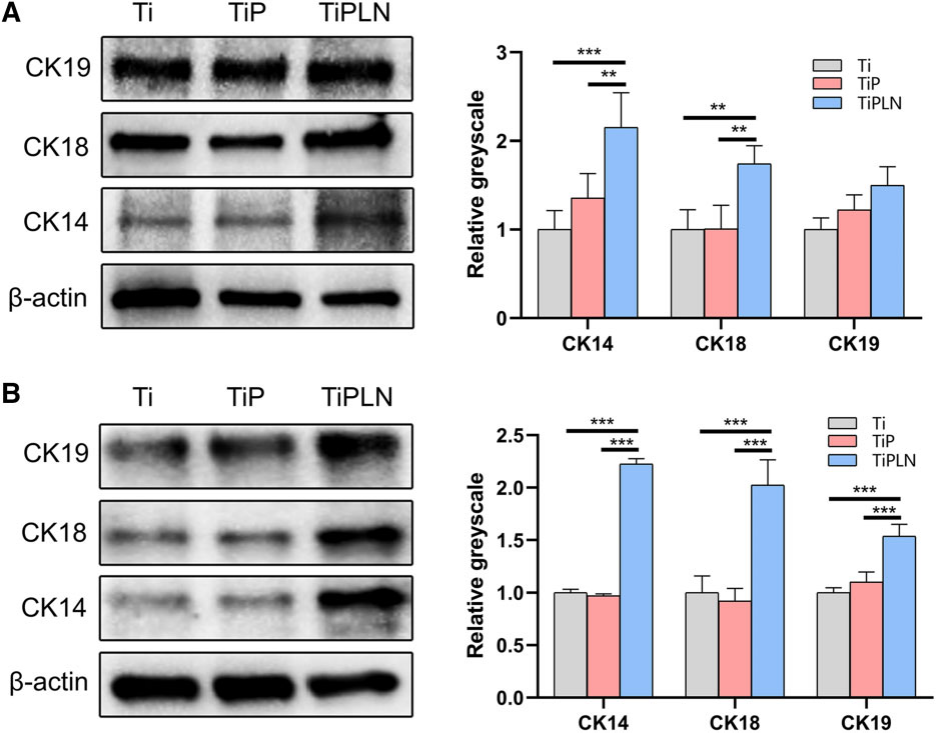
Western blot analysis and semi-quantification of GMSCs. (**A**) Western blot analysis and semi-quantification of CK14, CK18 and CK19 of GMSCs on Ti, TiP and TiPLN substrates at 3 days. (**B**) Western blot analysis and semi-quantification of CK14, CK18 and CK19 of GMSCs on Ti, TiP and TiPLN substrates at 7 days (**P <* *0.01; ***P <* *0.001).

Cytokeratin generally contains the epidermal cytoskeleton and performs important functions in the mechanical stability and integrity of epithelial cells [50]. The transdifferentiation of GMSCs into keratinocytes was marked by the expression of CK14, CK18 and CK19. As shown in Fig. 9, the expression of CK14, CK18 and CK19 on TiPLN was higher than that on Ti and TiP, although the expression of CK19 had no significant difference on Day 3, suggesting that TiPLN can induce the transdifferentiation of GMSCs into epithelial cells. The microenvironment around stem cells, including the recognition of ECM proteins, growth factors and the physical characteristics of substrates, is crucial to determining stem cell fates [51]. Shibata *et al.* [52] have found that the LN332 E8 fragments promoted human-induced pluripotent stem cells transdifferentiation into corneal epithelial cells. Lee *et al.* [53] have shown that laminin and MSCs had a synergistic effect on the regeneration of the tracheal mucosal epithelium. Our data provide the first evidence that LN332 can enhance the transdifferentiation of GMSCs into epithelial cells after loading onto the implant surface, indicating that GMSCs might participate in the peri-implant soft sealing by transdifferentiation into epithelial cells.

## Conclusion

In the study, we successfully constructed a PDLLA-LN332 composite coating by a method similar to LBL, which displays staged LN332 release for as long as 28 days. The coating promotes the expression of keratinocyte HD-related molecules, including integrin α6β4, LN332 and plectin, through the classical PI3K-Akt signaling pathway and benefits the adhesion, migration and proliferation of keratinocytes. In addition, the coating can promote the adhesion, proliferation and epithelial differentiation of GMSCs.

## Supplementary data

Supplementary data are available at *REGBIO* online.

## Funding

This work was supported by the National Natural Science Foundation of China (No. 81970971) and Shaanxi Key Research and Development Program (No. 2022SF-179).

*Conflicts of interest statement*. The authors declare no conflict of interest.

## Supplementary Material

rbac054_Supplementary_Data
