# Supplementary material for: Laminin 332-functionalized coating to regulate the behavior of keratinocytes and gingival mesenchymal stem cells to enhance implant soft tissue sealing
Source: Regen Biomater. 2022 Aug 2;9:rbac054. doi: 10.1093/rb/rbac054 (PMC9438747; doi:10.1093/rb/rbac054)
Supplement: rbac054_Supplementary_Data [file rbac054_supplementary_data.zip › Supplementary file.docx]

**Supplementary Table 1. Antibodies used for WB and IF**

| Antibody | Source | Identifier |
| --- | --- | --- |
| Integrin α6 antibody | GeneTeX | Cat# GTX100565 |
| Integrin β4 antibody | GeneTeX | Cat# GTX75041 |
| Laminin332 antibody | R&D Systems | Cat# MAB21441 |
| Plectin antibody | Cell Signaling Technology | Cat#12254 |
| PI3K antibody | Affinity Bioscience | Cat#AF6241 |
| p-PI3K antibody | Affinity Bioscience | Cat#AF3241 |
| Akt antibody | Affinity Bioscience | Cat#AF6261 |
| p-Akt antibody | Affinity Bioscience | Cat#AF0016 |
| Cytokeratin 14 antibody | Abcam | Cat# ab7800 |
| Cytokeratin 18 antibody | GeneTex | Cat# GTX105624 |
| Cytokeratin 19 antibody | Abcam | Cat# ab76539 |
| β-actin antibody | GeneTeX | Cat# GTX109639 |
| GAPDH antibody | Affinity Bioscience | Cat#AF7021 |
| CD14 | BioLegend | Cat#325606 |
| CD34 | BioLegend | Cat#343506 |
| CD44 | BioLegend | Cat#338808 |
| CD45 | BioLegend | Cat#304008 |
| CD90 | BioLegend | Cat#328110 |
| CD105 | BioLegend | Cat#323206 |
| HRP-conjugated anti-rabbit secondary antiboy | Beyotime | Cat#A0208 |
| HRP-conjugated anti-mouse secondary antiboy | Beyotime | Cat#A0216 |
| Fluorochrome-conjugated anti-rabbit secondary antiboy | GeneTeX | Cat# GTX213110-05 |
| Fluorochrome-conjugated anti-rabbit secondary antiboy | GeneTeX | Cat# GTX213111-04 |
