# Supplementary figures and images for: Laminin 332-functionalized coating to regulate the behavior of keratinocytes and gingival mesenchymal stem cells to enhance implant soft tissue sealing
Source: Regen Biomater. 2022 Aug 2;9:rbac054. doi: 10.1093/rb/rbac054 (PMC9438747; doi:10.1093/rb/rbac054)

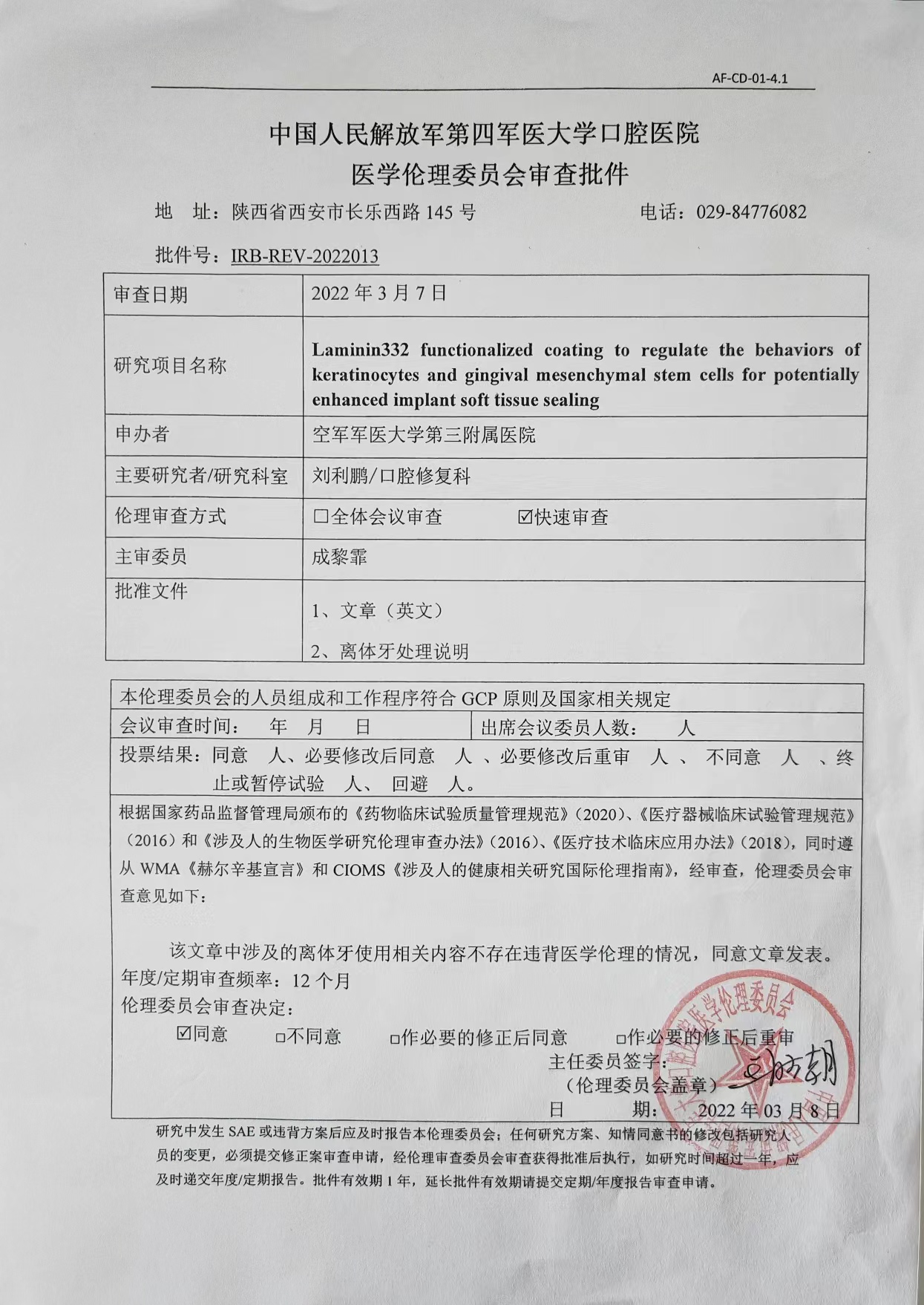

Supplement: rbac054_Supplementary_Data [file rbac054_supplementary_data.zip › Ethics approval and consent to participate.jpg]
